# A new perspective of Aristotle’s theory of vision: analysis of the “psycho-physical” intertwined mechanism of vision

**DOI:** 10.3389/fpsyg.2023.1264335

**Published:** 2023-10-17

**Authors:** Qi Zhao

**Affiliations:** Department of Philosophy, School of Marxism, Northeastern University, Shenyang, China

**Keywords:** Aristotle, vision, color, transparent substance, light

## Abstract

Aristotle’s visual theory plays a pivotal role in *De Anima*, he specifically analyzes three fundamental elements required for visual activities, namely, color, transparent substance, and light. Color moves and limits transparent substance, thereby transforming transparent substance from potentiality to actuality through light. However, there is a debate between Physicalism and Spiritualism as to the specific implementation of the visual activity. Through the intertwined mechanism, Aristotle’s theory of vision can be clarified. The visual activity is neither purely psychological nor purely physical, it is the “psycho-physical” intertwined mechanism. This is why Aristotle’s visual theory is closely related to contemporary visual psychology.

## 1. Introduction

Aristotle begins his analysis of five individual senses, namely vision, hearing, smell, taste, and touch, from *De Anima* II.7 to II.11. Hicks (1907) questions the order of discourse on individual senses in *De Anima*. The rule established by Aristotle for the order is to start with the most fundamental sense, namely touch. Nevertheless, Aristotle places vision at the forefront of his discourse, violating the rule in *De Anima*. In the view of Shields (2016), it is necessary to place vision at the forefront of Aristotle’s theory of sensation, as it is a paradigmatic sense compared to other individual senses. However, Shields fails to demonstrate why vision is paradigmatic compared to other individual senses, he only makes clear his stand. Bynum (1987) supplements this by stating that the reason why vision is paradigmatic is because Aristotle’s visual theory is the most detailed in *De Anima*. Nonetheless, the question about the exemplary vision is as follows: are we only aware of an object’s color by purely conceptual mind when we see it, or are our eyes physically changed by color? In order to resolve this dilemma, the academia has debated between Physicalism and Spiritualism. Scholars represented by Slakey (1961), Sorabji (1974), Broadie (1993), and Johansen (1997) hold a Physicalist interpretation. When eyes look at color, the Physicalists think that eyes can be colored. However, scholars represented by Philoponus (2005), Ebert (1983), Burnyeat (1995a,b), and Scaltsas (1996) hold a Spiritualist interpretation, as follows: the visual activity does not involve the physical process. Only the psychological state changes during the process, there is no physical impact on our eyes. Both of these views are misunderstandings of Aristotle’s visual theory. If we regard vision as a purely physical activity, it loses the efficient cause of vision. According to Physicalist interpretation, only physical activities occur in visual behaviors, but this explanation shows that Physicalists overlook the psychological form of vision. Aristotle points out in *Metaphysics* that there is no pure matter in actuality, and it must be a form-involving matter. The form is crucial for the matter, namely “what the process towards health begins from” (Aristotle, 1994). The efficient cause is a term used by Aristotle, and it is easier to understand if we paraphrase it as “motive force.” This is obvious for vision, if without the visual ability, eyes would not be able to see color. If eyes are only colored red, there would be no visual activities. Visual activities are psychological behaviors, but “coloration” is only a purely physical behavior. Therefore, when we describe eyes, we refer to the eyes with the visual ability, and there are no purely physical eyes for our vision. Consequently, the psychological ability is the efficient cause of “coloration.” As for the explanation of Spiritualists, they overlook the crucial role of physical behaviors in psychological activities, the psychological activities should be regarded as “psycho-physical” intertwined activities. If psychological activities are not based on physical activities, then psychological activities cannot be achieved. This paper will follow Charles, in his view, the matter and the form cannot be separated from each other. The matter is a form-involving matter, and the form is a matter-involving form. Therefore, no pure form or pure matter exists (Charles, 2021). However, Charles’ analysis concentrates on the macroscopic relationship between the soul and the body in *De Anima* II.1–3. Despite the fact that Charles explores the visual theory in *De Anima* II.7, he does not implement the basic principle of the “inextricably psycho-physical activity” in this chapter. By contrast, this article argues that these three elements are all “psycho-physical” intertwined components. Moreover, Charles also believes that transparent substance serves as the medium between the visual organ and the visual object. In Aristotle’s *De Anima* II.7, transparent substance is not only the medium between the visual organ and the visual object, but also “within” the visual organ, and even “within” the visual object. In addition, Charles analyzes light from “illumination,” but light’s impurely physical nature is precisely a necessary link in Aristotle’s visual theory. Given that this situation, this article aims to demonstrate the “psycho-physical” intertwined mechanism in Aristotle’s visual theory.

## 2. The mechanism of visual activity and its difficulties

Aristotle (2016) points out in *De Anima* II.5 that the actualization of individual senses is based on possessing external sensory objects. Consequently, the analysis of vision is based on expounding the visual object. Accordingly, to some extent, the external object has visibility. We should analyze “visibility” into two concepts, one is color, another is the unnamed luminous element, for which in Aristotle’s view, there is no corresponding term, and later scholars refer to it as phosphorescence. The former is in the light, and it contains intrinsic visibility; The latter is in the dark. The phosphorescence is seen through its own light, as in the examples of fungus, skin, insect scales, and fish eyes (Aristotle, 2016). Color is not necessarily visible by its own purely conceptual definition, but rather serves as a “psycho-physical” intertwined element. The so-called “purely conceptual definition” refers to the definition of color solely from a purely psychological perspective, and the visibility of color can only be analyzed through a purely psychological dimension. The reason why it is called “purely conceptual” is because the definition of color lacks a physical dimension, and the “pure conceptual” refers to the fact that color only exists in our subjective mind. Color should be understood as a “psycho-physical” intertwined component. If we only start with a purely conceptual definition, it indicates that we fail to analyze the essential meaning of color. In short, color is not a purely psychological component, but a combination of the psychological component and the physical component. The effects of color on transparent substance are as follows: (1) Color triggers movement of transparent substance, thereby making the corresponding object visible (Aristotle, 2016); (2) Color limits transparent substance (Aristotle, 1991).

Transparent substance serves as a medium for visual activities. In a broad sense, transparent substance can be referred to as an “ontological medium” because the process of vision relies on transparent substance, not only are our eyes based on transparent substance, but also every visual object has transparency, even if it is a completely solid entity. It can be seen from this that transparent substance is the medium that maintains coherence between the visual organ and the visual object. In a narrow sense, transparent substance should be regarded as a medium between the visual organ and the visual object, such as water or air between our eyes and colored objects. Regardless of the broad sense or the narrow sense, transparent substance is not the purely physical entity outside of specific elements, but rather the entity that possesses the “indwelling nature” of different elements (Aristotle, 2016). This indicates that transparent substance is not a purely physical medium, but rather a “psycho-physical” intertwined medium. Granted that transparent substance is regarded as a purely physical entity, then Aristotle must be mistaken for a Pre-Socratic philosopher. Even though both air and water are transparent, our eyes are composed of water, not air, for it is absurd to think that air is the matter of eyes. Although water is the component of the visual organ, it is a metaphorical concept, not the drinkable water in a pure sense. The so-called metaphor is not completely abstract, but is a “psycho-physical” intertwined activity in the “definition.” This means that, when referring to water in the context of the matter, it has no purely physical characteristics but can receive the characteristics of physical substance.

Moving and limiting visual objects through color can only make transparent substance a potential medium, and only through light can the potential medium be transformed into the actual medium. Accordingly, light should be understood as the “transformation” or the “illumination” from potentiality to actuality, and this visual activity is a “psycho-physical” intertwined process. This is because light is not only the activity of activating transparency but also enables color to be seen, “What is seen in light is color, which, accordingly, is not seen without light” (Aristotle, 2016). Aristotle analyzes the source of light: Light comes from fire. As a consequence, fire has its unique characteristics. Water, air, and earth can be seen in light, but only fire can exist in both light and dark, because fire is the source of light and not limited by light. Moreover, as a luminous entity, transparent substance can also be seen in the dark. Notwithstanding, it should be noted that although light comes from fire, it does not possess the characteristics of fire (Aristotle, 2016). Therefore, Aristotle (2016) asserts that light is not a purely physical form. It is only the “actuality of the transparent.”

However, due to the simplicity and ambiguity of Aristotle’s visual theory, there are some confusions in *De Anima* II.7. First, Aristotle (2016) believes that there are two visible forms, namely color and phosphorescence, the color being the focus of his argument in *De Anima*. Nonetheless, he also harbors the idea that the visibility is only an innate attribute of color, not a purely conceptual definition of color. Is there a physical definition of color beyond the purely psychological definition? Second, despite the fact that Aristotle reveals the fundamental difference between color and phosphorescence, he fails to demonstrate the intrinsic meaning of phosphorescence. The explanation for why we could see phosphorescence should be discussed elsewhere (Aristotle, 2016). The above two questions can be reduced to one question: Is the visual activity a purely physical behavior or a purely psychological behavior? This is the debate between Physicalism and Spiritualism in academia on Aristotle’s visual theory.

Is transparent substance a purely psychological medium or a purely physical medium? Transparent substance is invisible by itself and should be made visible by the color of other physical objects, thereby becoming a visual medium in the sense of potentiality. This phenomenon induces readers to interpret transparent substance as something accidental. Moreover, are only transparent elements such as air and water based on transparent substance? If so, then transparency cannot belong to all visual objects, other fundamental elements such as fire and earth do not have transparency. However, the absurd consequence is that our eyes would inevitably only be able to see a few elements and objects in light, but not all elements and objects, which goes against the function of vision in *De Anima* II.7. Furthermore, would the attributes of transparent substance change when color limits it? Johansen (1997) believes that an affection of the eye. Does transparent substance essentially lose transparency after being colored? Strictly speaking, the behavior of “coloration” can only be understood in terms of symbolic or metaphorical meaning, rather than our eyes being colored in a purely physical sense. The so-called “symbolic or metaphorical” is not completely abstract, but is a “psycho-physical” intertwined activity in the sense of “definition.”

There are also confusing difficulties to “light.” Is it a purely physical element like fire? Supposing that light comes from fire, would light cause a purely physical effect on the colored object? If there is a physical effect, would it cause two visual objects to occupy the same position? Moreover, Aristotle regards light as the implementation of transparency, would the attributes of transparent substance change during vision’s implementation process? The meaning of light in the verb sense can be divided into two situations: (1) light is the activity of “illumination.” The reason our eyes need light is because we cannot see a color in the dark. Only through the illumination of light can color has visibility, and vision can actualize its ability to “see color.” (2) Aristotle’s concept of light in *De Anima* is also an ontological metaphor, the metaphorical meaning is “transformation.” Specifically, Aristotle demonstrates the difference between potentiality and actuality. Granted that the visual ability is not utilized, it is only a potentiality. Only when the visual ability is being utilized is it the actual state of vision. In Aristotle’s view, light is a metaphor for the transformation of vision from potentiality to actuality. Consequently, The meaning of metaphorical light is “transformation.” The verbalized implementation activity of light can be divided into two types: The literal “illumination” and the metaphorical “transformation.” Nonetheless, does the implementation activity cause light to lose its ontological foundation? Many questions about color, transparent substance, and light revolve around controversies over psychological and physical activities in *De Anima* II.7. This article meticulously elaborates on the fundamental elements of vision from “psycho-physical” intertwined mechanism.

## 3. The essence of color and its psycho-physical interaction on visual activities

According to Aristotle, the reason why color is visible is not because of its purely physical characteristics, but because it is the concept relative to vision, so the essence of color is visibility. Put it bluntly, the essential definition of color depends on whether it can be seen, not it’s purely physical attribute. Therefore, we should crystallize color from the perspective of visual psychology. However, the concept of color is not a purely psychological form. The essential meaning of color is a non-purely psychological form, it involves physical substance in the definition. From this, it can be seen that the definition of color is complicated, and its psychological definition is mixed with the non-purely physical definition. In short, we can take an attitude that color is a “psycho-physical” intertwined component in Aristotle’s visual theory.

Although color is the “psycho-physical” intertwined component, it cannot be equated with the colored object. Given that the distinction between the two, Themistius (2013) refers to both color and colored object as “visible object,” obviously confusing physical substance and its form. According to Aristotle, color is the physically psychological form *in De Anima*, rather than the colored object in a physical sense. Simplicius (2013) maintains that color cannot be equated to the colored object. Simplicius’ view is a correction of Themistius’ erroneous position. To be precise, color is not the “visual object,” but only the “form” of the visual object, this is because vision “is what is capable of receiving perceptible forms without the matter” (Aristotle, 2016). We can also demonstrate the distinction between color and colored object from the difference between the two-dimensional plane and the three-dimensional solid. Why does color is the form of its corresponding colored object? According to Aristotle’s *Sense and Sensibilia*, this is because color only has features of the two-dimensional plane, it does not penetrate the three-dimensional structure of colored objects but rather lies on their surface (Aristotle, 1991). Specifically, color limits the transparency of a colored object on its surface, thereby affecting the transparency of a colored object. Therefore, color is a mixture of proportions in the two-dimensional plane, such as the mixture between white and black (Barker, 1981).

We could see a color in light, while the phosphorescence can only be seen in the dark. The essential relationship between color and phosphorescence can be analyzed through “psycho-physical” intertwined mechanism. Based on the intertwined mechanism between psychological activities and physical activities, the difference between color and phosphorescence can be explained. The essential difference between color and phosphorescence depends on the “light” on which they rely. Only through light can transparent substance become an actual medium. We should understand the phosphorescence in the sense of “brightness,” rather than the light that emitted from fire in a purely physical sense. This brightness is only an inherent characteristic of phosphorescence. Not only is it not light, it also masks the color that the colored object should possess. If phosphorescence is believed to emit light in the dark, it seems that the dark is a purely physical space opposite to light. This conflicts with Aristotle’s original intention in *De Anima* II.7, as the dark is a non-purely “psycho-physical” space. The form that glowing in the dark cannot be viewed as color, but this does not influence its visibility. Color is visible, but this does not mean that only color can possess visibility, phosphorescence can also possess visibility, for it is a non-purely “psycho-physical” intertwined element.

## 4. The “psycho-physical” mechanism of transparent substance

Transparent substance cannot be seen in itself, but must be made visible through color. Does transparent substance exist as an accidental condition of the visual object? If transparent substance is a purely physical component, then it is accidental in visual activities, as vision is a “psycho-physical” mechanism. If it is a “psycho-physical” intertwined component, such as eyes, then it is essential in visual activities. Transparent substance cannot be understood as an accidental object in Aristotle’s visual theory. According to Aristotle (2016), the transparent substance should not be viewed as literally transparent air or water, but rather the entity that has transparency as an indwelling nature among visual objects. The term “indwelling nature” here refers to the fact that transparency, as a common essence of elements like air and water, exists in a three-dimensional substance compared to the two-dimensional color on the surface of visual objects. This indwelling nature indicates that transparency is a “psycho-physical” intertwined nature, showing that this medium is an inextricably “psycho-physical” component. Not only do visual objects have transparency, but our eyes also have the transparency. Therefore, eyes are also based on transparent substance. If one attempts to remove a transparent medium and directly place the visual object on our eyes, the visual activity would inevitably fail (Aristotle, 2016). Transparent substance is the material that makes up eyes, it can be said with certainly that that eyes have the purpose and motivation to see the visual object, so transparent substance is a psychological matter, which can also prove the non-purity of transparent substance in the intertwined mechanism of “psycho-physical.”

Is transparency only present in some elements such as air or water? or is transparency present in all elements and all corresponding physical objects? If transparent substance is a pure matter, it can only be possessed by certain visual objects. If it is a “psycho-physical” intertwined component, then all visual objects have transparency in the sense of “psycho-physical” mechanism. From perspective of Polansky (2007), there are five fundamental elements: water, fire, air, earth, and ether. All elements have transparency, not just a few elements like water and air. Polansky’s opinion aligns with Aristotle’s visual theory in *De Anima* that not only elements like air and water have transparency, but also everything has transparency, to varying degrees. Transparent substance can receive the characteristics of any other objects, but it does not have any characteristics in itself (Aristotle, 2016). This means that transparent substance does not contain purely physical materials. However, it cannot be ruled out that transparent substance includes matter in its non-pure definition. Although it is believed that transparent substance refers to air or water, it does not refer to purely material component, but rather to the “psycho-physical” nature of elements such as air or water. However, transparent substance must be an element that is involved in metaphorical air or water, namely non-physical air or water, otherwise transparent substance lacks the motive force for implementation. Transparency belongs to all elements and objects composed of elements, as transparent substance serves as a visual medium and is indispensable for visual activities.

Although transparent substance is limited by color, its own attributes do not undergo indispensable changes, as transparent substance is only colored in the dimension of the non-pure definition, not physically colored in the pure sense. In this case, the “psycho-physical” activity is an intertwined behavior only in the sense of definition. For transparent substance, color only exerts an influence on a two-dimensional plane from the outside. It cannot penetrate the interior of transparent substance, resulting in fundamental changes of transparency. However, transparent substance is only visible through color. It is not purely material features. During this visual process, transparent substance can preserve its own nature in a “psycho-physical” intertwined sense. If it frequently changes, it cannot serve as the medium for visual activities. In this case, Johansen’s interpretation of Aristotle’s visual theory is open to dispute. He says that “seeing is an affection of the eye simply because the eye is understood as a potentiality to see” (Johansen, 1997). Slakey (1961) also takes a similar attitude. Burnyeat’s critique of such views is representative, he distinguishes between the following two types of changes: (1) the change happens in contraries, such as someone becoming knowledgeable having been ignorant. (2) The visual ability only transforms from potentiality to actuality. In this process, there is no change in the physical sense (Burnyeat, 1995b).

## 5. The “psycho-physical” intertwined mechanism of light: the transformation of vision

The reasons for discussing the nature of light in this section are as follows: (1) Light is a paramount concept of visual theory and it is a key element of the visual activity along with color and transparent substance. The reason why light is necessary for visual activities is because without the “illumination” of light, eyes cannot see the form of the visual object. (2) The “illumination” activity of light is not only in the literal sense, but also in the metaphorical sense. Visual activities are not static, but dynamic behaviors of vision. The light in a metaphorical sense should be understood as a verbal “transformation,” that is, a transformation from potentiality to actuality. (3) Light is a “psycho-physical” intertwined element. It can be seen from (1) and (2) that regardless of whether in terms of literal “illumination” or metaphorical “transformation,” light is an essential component of the visual activity. Now that color is a “psycho-physical” intertwined element, vision is a “psycho-physical” intertwined activity, and we can conclude that light must be a “psycho-physical” intertwined element, otherwise it is impossible to “transform” or “illuminate.”

How does light exist? Aristotle (2016) believes that light is the “actuality of transparency.” Does this activity of actualization exist as a purely physical behavior? Aristotle’s description in *De Anima* II.7 easily leads readers to regard the illumination of light as a purely physical activity, but this is a misreading of Aristotle’s visual theory. It can be determined that as light is an actual medium of vision, it cannot be regarded as a purely physical element, but rather a “psycho-physical” intertwined element. Hamlyn (1968) believes that even though fire is the initial source of light, we cannot view illumination as a purely material activity, while the illumination is a necessary condition for our eyes to see color, otherwise we lose the ability of vision in the dark. Although light has no physical characteristics that can be separated in a pure sense, it involves non-purely physical activities, whereby “psycho-physical” activities are intertwined in conceptual definition. Themistius, drawing on three aspects, denies that light includes purely physical features that can be separated in physical space. First, in case that light contains purely physical features, it must be regarded as a purely physical entity occupies a physical position, and transparent substance also serves as a material entity. Obviously, this is a wrong outcome. Second, supposing that light produces purely physical effects, then wind can blow the light that contains purely physical effects. Certainly, this is a misunderstanding of light. Third, if light produces purely physical effects, it moves according to its own pure nature. However, Aristotle’s theory of light is timeless (Themistius, 2013). How should we understand the position of Themistius? According to Aristotle’s definition in *Physics* and *Metaphysics*, “time” is a way of measure, excluding the psychological dimension. Nevertheless, light is not a purely physical element in Aristotle’s *De Anima*, and its illumination is also not a purely physical activity. Owing to light is a necessary condition for visual activities, vision is a “psycho-physical” intertwined activity, we can conclude from this that light must be a “psycho-physical” intertwined element. From a metaphorical perspective, light is timeless because it is only a symbolic or metaphorical description. It can be seen from this that light is timeless, as it does not possess purely physical characteristics. Nevertheless, the literal meaning of “illumination” is still in non-purely physical time.

Light does not include purely physical characteristics, how can it trigger physical change in the attributes of transparent substance? Due to the “psycho-physical” intertwined activity of light, illumination is the process of transforming the potential medium into the actual medium, so we should also analyze the light in the sense of metaphorical “transformation.” It should be noted that such a transformation would cause a fundamental change in the attributes of transparent substance. This psychological transformation essentially involves non-purely physical components in its definition, otherwise there would be a lack of efficient cause to actualize psychological activity. Aristotle believes that light is the manifestation of fire, and that light is not a purely physical entity because two physical entities cannot be in the same place. In addition to the potentiality in the dimension of metaphorical transformation, light also serves as a concept of actuality. Philoponus (2005) holds that actuality even more important than potentiality.

## 6. The mutual foundation of visual elements

We have analyzed three fundamental elements of the visual ability in previous sections, namely, color, transparent substance, and light, all of which are “psycho-physical” intertwined elements. Due to the fact that the visual behavior is composed of three fundamental elements, it is a “psycho-physical” intertwined activity. Nonetheless, we should investigate the interrelationship between color, transparent substance, and light, it is a necessary condition for understanding visual activities. This paper has analyzed three constituent elements of color, transparent substance, and light, but this analysis is more from a static dimension and has not yet established a dynamic relationship between the three. Nevertheless, the visual activity is not just concerning potentiality, but more importantly regarding actuality, so we need to further analyze the dynamic process of visual activities. This is why we analyze the dynamic relationship between color, transparent substance, and light. In case that this paper only confines the three elements to conceptual analysis, we cannot establish a dynamic process of vision. Only by establishing a mutual foundation between color, transparent substance, and light, and analyzing the dynamic interrelationships among the three, can the visual behavior shift from the static concept to the dynamic activity. This is in line with Aristotle’s analysis of actuality in *De Anima* II.5. The concept of actuality has double dimensions. First, it is the “first actuality,” which means that vision only has the ability to see color, but not in the process of implementation; Second, it is the “second actuality,” which means that vision not only has the ability to see color, but also in the dynamic process of implementation. Therefore, the discussion of the interrelationship between color, transparent substance, and light is a necessary condition for the second actuality of the visual ability.

Aristotle’s argument on the fundamental elements of vision is not fragmented, but rather constitutes a structural relationship in the sense of dynamicity whereby color, transparent substance, and light are all in a mutually foundational relationship. The first type is the mutual foundational relationship between color and transparent substance. Color moves and limits transparent substance, making it visible and transforming it into a visual medium. It should be noted that color can only move transparent substance, and the “psycho-physical” intertwined process only exists in a non-purely conceptual definition. Color is the form of the colored object. It can only trigger a “non-standard” change rather than a purely physical change. The non-standard change is a “psycho-physical” change in the non-purely conceptual definition, for the transparent substance has no purely physical or purely psychological characteristics. Everson (1995) harbors the idea that color is not the form of the visual object in contemporary philosophy of mind, but rather the formal cause of the sensory object. Only when transparent substance exists in the dimension of “psycho-physical” intertwined processes can color move and limit them, otherwise color loses the ontological foundation.

Color and light are also in a mutually foundational relationship. Color makes light possible as a “psycho-physical” illumination, otherwise light would be a purely psychological form. Only through color can light has an actual visibility. Therefore, color is an essential condition for light to actualize transparency. This proves that there is an essential difference between Aristotle’s visual theory and the theory of intention. Aristotle acknowledges the existence of the external physical object, otherwise color would be impossible to actualize a non-purely conceptual activity. A further advantage is that light is a necessary prerequisite for the color to become inherently visible, otherwise color would be a purely physical matter. It is only when color already in the process of implementation that it can be considered as having this potentiality. Broadie (1993) observes profoundly that the perceptual object is essential. Only when light is actualized can color move and limit on the surface of the visual object.

Light and transparent substance lay the foundation for each other in visual activities. Transparent substance is the foundation for light to carry out visual activities because light acts on transparent substance, otherwise light cannot be actualized; In addition, light is the fundamental premise of transparent substance. Transparent substance has first to be in the process of actualization, to be considered a visual medium in the sense of potentiality. Most scholars regard vision solely as an activity of seeing, while Caston (2002) notes the double mechanism of Aristotle’s visual theory. The visual activity is not only simply the behavior of seeing, but also includes the behavior of “*to krinein.*” Therefore, visual activities can be divided into two levels: (a) how to see color, and (b) “how to perceive that we see” (Aristotle, 2016). Ebert (1983) believes that quite a few English translations translate “*to krinein*” as “judgment,” such as Hamlyn’s translation (Hamlyn, 1968), which confuses sensation and reason because judgment behavior is a rational ability, and sensory behaviors can only be understood as “distinguish,” which is a behavior without making judgments regarding right or wrong. Vision is not exclusive to human, but rather an ability possessed by the vast majority of animals. Animals other than humans have vision, but they do not possess reason, because the reason is unique to humans. However, “judgment” is a rational behavior, as the judgment is based on the copula “is.” As an example, animals can only distinguish between red and green, but cannot conclude that “red is a color different from green.” Animals simply distinguish between red and green, there is no distinction between right or wrong. This is because animals lack language, they cannot use the copula “is” to make judgment, let alone induce from individual red and green to the universal concept of “color.” Consequently, “*to krinein*” in Aristotle’s *De Anima* is the behavior of “distinguish.”

## 7. Connecting Aristotle’s theory of vision with later and present theories of vision

Aristotle’s visual theory has caused significant impacts on contemporary Functionalism. According to Aristotle’s visual theory in *De Anima* II.7, the visual organ and its visual ability are inextricable, whereby the visual ability is the form of the visual organ, and the visual organ is the matter of the visual ability. Aristotle’s visual theory can be traced back to the concept of the soul in a macro sense. Animal’s soul has multiple functions, and vision is one of the most crucial functions. For this reason, in Johnston’s view, we should shed light on the soul “in terms of its powers” (Johnston, 2011). The resulting issues are as follows: can Aristotle’s visual theory be revitalized? How should we expound the contemporary significance of Aristotle’s visual theory?

Some contemporary scholars aim to connect Aristotle’s visual theory with contemporary Functionalism, represented by scholars such as Nussbaum (1978), Nussbaum and Putnam (1995), and Green (1998). According to the explanation of Functionalism, the visual ability in Aristotle’s *De Anima* cannot be reduced to a purely physical activity that are no different from the coloration. However, as a function, vision should be the function of eyes. Therefore, vision cannot exist independently of eyes. Significantly, can contemporary Functionalism really maintain an independent position between Physicalism and Dualism? Green denies the possibility that Functionalism can insist this independent status, believing that it is a transformation of Physicalism, only rising to dominance in the 1960s when Physicalism and Behaviorism lost support (Green, 1998). We should be cautious about Green’s viewpoint. As a matter of fact, the best explanation is that Physicalism is compatible with Functionalism. According to Functionalism, the relationship between the visual ability and the visual organ in Aristotle’s *De Anima* can be analogized to the relationship between software and hardware: This example has been used by many Functionalists. The function of software must be based on hardware to operate. However, software does not require specific and individualized hardware, so the material requirements for hardware are broad, and the correspondence between software and hardware is not unique. Similarly, the actualization of the visual ability should be based on the visual organ. Nevertheless, the visual ability does not require the specific and individualized visual organ, thus the correspondence between the visual ability and the visual organ is not unique. From this, it can be seen that according to Functionalism, the matter plays a fundamental role in visual activities. Without physical substance, the visual ability cannot be actualized at all. Functionalism is similar to Physicalism, because the two all hold that the matter plays a crucial role in visual activities. It should be emphasized that there is still a fundamental difference between the two: Functionalism believes that physical substance plays a fundamental role in visual activities, but does not negate the existence of the psychological behavior. However, according to Physicalism, when the visual activity occurs, there is no change in our psychological state, so vision is not a psychological behavior. Contrarily, the visual activity is only a physical behavior. For instance, they all hold the view that when we look at red with our eyes, our eyes would be colored red. Apart from this behavior of “coloration,” there would be no psychological activities, such as emotional or conscious changes.

Second, Functionalism is an anti-reduction position. To be precise, determining the eye’s essence is the visual activity that cannot be reduced to physical activity, rather than the eye itself, Nussbaum is a representative anti-reductionist (Nussbaum, 1978). Finally, Functionalism is an Isomorphism, it is the core viewpoint held by Functionalists. For contemporary Functionalists, Aristotle’s concept of soul should be defined as the function that cannot exist without matter. As a function of the soul, vision cannot be separated from the visual organ. However, what Nussbaum and Putnam (1995) say concerning material is only a universal concept of the material, whereby, for individual type of the material, vision can serve as the function of different eyes. Therefore, the foundation of a visual form may not necessarily be composed of individual eyes, and this material composition can only be accidental. It can be seen from this that Putnam holds a Functionalist stance. The relationship between the visual ability and the visual organ should be analyzed in Putnam’s textual context. Burnyeat summaries Putnam’s view that the individual body is unnecessary for Functionalists. Consequently, Burnyeat harbors the idea that “Aristotle’s conception of the material or physical side of the soul-body relation is one which no modern Functionalist could share” (Burnyeat, 1995b). We should dialectically view Burnyeat’s summary: on the one hand, Burnyeat keenly notes that Putnam’s explanation can be classified as Functionalism. For Functionalists, the visual ability does not require the individualized visual organ. Accordingly, Burnyeat accurately recognizes the fundamental difference between Aristotle’s philosophy of mind and contemporary Functionalism. On the other hand, based on Burnyeat’s summary, we still need to recognize that Putnam is still somewhat different from traditional Functionalists, as he takes the attitude that there is an essential difference between human’s vision and animal’s vision (Nussbaum and Putnam, 1995). Regarding the term “correspondence,” there is a difference between Putnam and traditional Functionalists. It should be emphasized that Putnam recognizes to some extent the “non-extensive” correspondence between the function and the physical substance, and the relationship between the two is not completely arbitrary. From this, it can be seen that Putnam’s interpretation and Aristotle’s theory of vision are to some extent compatible, as Putnam is a “special” Functionalist. In summary, the Functionalist interpretation is that there is no uniqueness of correspondence between the vision and the visual organ. Putnam’s term “correspondence” in its context is a limited innovation of Functionalism, recognizing that the relationship between the visual function and the visual organ corresponds in terms of species, but he fails to realize the uniqueness of the correspondence between the visual organ and the visual ability, that is, a certain visual ability can only correspond to a unique visual organ.

Aristotle’s visual theory is closely related to contemporary Structuralism, Behaviorism, and Gestalt Psychology. Contemporary psychology has two influential schools, namely Structuralism and Behaviorism. Contemporary psychologists represented by Wundt (1902), Titchener (1908), and Pettit (1977) hold a Structuralist perspective. In their view, the research topic of psychology is not about a perceptual behavior, but rather about human subjective consciousness. From this, it can be seen that structuralists argue that the topic of vision is only concerned with consciousness or mind. However, psychologists represented by Watson (1919), Skinner (1974), and Leigland (1992) hold a Behaviorist perspective, believing that the object of psychology is not mind or consciousness, but rather the objectification of human behaviors. Structuralism and Behaviorism correspond to the Physicalist interpretation and Spiritualist interpretation of Aristotle’s visual theory, respectively, both Structuralism and *De Anima*’s Spiritualist interpretation believe that visual behaviors are conscious activities, that is, as Aristotle’s visual theory in *De Anima* II.7, when we look at the color of something, we are only aware of it. Behaviorism corresponds to the Physicalist interpretation of Aristotle’s visual theory, which states that when we see color, our eyes only engage in physical activities. Therefore, Aristotle believes that when we look at red, our eyes would be colored red. Due to Aristotle’s theory of vision being a “psycho-physical” intertwined mechanism, Behaviorism and Structuralism only consider one side of Aristotle’s visual theory and cannot fully reflect the richness of *De Anima* II.7. Compared to Behaviorism and Structuralism, Aristotle’s visual theory is more similar to contemporary Gestalt psychology. Gestalt psychologists, represented by Köhler (1929) and Wertheimer (1982) believe that neither Behaviorism nor Structuralism can properly describe visual phenomena. This means that the visual activity has the double dimensions of physical and conscious activities. This viewpoint is closely related to Aristotle’s theory of vision. According to Aristotle, the physical activity of vision is not a pure activity, but a psychologically physical activity; The psychological activity is not a pure activity, but a physically psychological activity. This reveals that Aristotle’s theory of vision is closely related to contemporary psychology. Concerning contemporary behaviorism and structuralism, these two perspectives are closely related to Aristotle’s theory of vision. Compared to Behaviorism and Structuralism, Aristotle’s visual theory is more closely related to contemporary Gestalt psychology.

Aristotle’s visual theory has a certain degree of connection with present visual theory. According to the contemporary theory of vision, the visual activity is the sum of sensation and perception. In essence, visual activities allow us not only to receive the external object and its related sensory experience, but also to systematically integrate sensory experience to form an introspective consciousness. This contemporary perspective is intensely similar to Aristotle’s theory of vision. According to Aristotle, we are not only able to see color, but also aware of the ongoing visual activities, Therefore, we have self-awareness in visual activities (Aristotle, 2016). For Aristotle, seeing color is a sensory behavior, while self-consciousness is a perceptual behavior. It should be noted that Aristotle’s visual theory does not reach the level of contemporary visual science due to the limitation of the times. As an illustration, Aristotle believes that eyes are based on transparent substance, but could not recognize the specific structure of eyes, such as the cornea, iris, pupils, and retina. The above discussion highlights that Aristotle’s visual theory has a similar perspective to contemporary psychological schools. This is not only reflected in the close relationship with Functionalist interpretation but also in the close correlation with Behaviorism, Structuralism, and Gestalt psychology.

## 8. Discussion

Aristotle’s theory of vision not only provides an individualized interpretation of the “potentiality-actuality” in *De Anima* II.1–2, but also plays an exemplary role in the five individual senses in *De Anima* II.7–11. The visual object is mediated by transparent substance, which cannot be seen by itself. Transparent substance is moved and limited by color. This behavior can only make transparent substance potential, and only through light, can the potential medium transform itself into the true medium. This study is grounded in the “psycho-physical” intertwined mechanism. Color, transparent substance, and light are in a “psycho-physical” intertwined relationship. However, there are many difficulties in Aristotle’s discourse on three fundamental elements of visual activities. By solving these difficulties, not only can we understand the essential connotations of three elements, but also have a more comprehensive understanding of vision’s implementation, whereby vision is an intertwined activity.

## Author contributions

QZ: Visualization, Writing – original draft.
